# Discovery of Novel Small-Molecule Inhibitors of LIM Domain Kinase for Inhibiting HIV-1

**DOI:** 10.1128/JVI.02418-16

**Published:** 2017-06-09

**Authors:** Fei Yi, Jia Guo, Deemah Dabbagh, Mark Spear, Sijia He, Kylene Kehn-Hall, Jacque Fontenot, Yan Yin, Mathieu Bibian, Chul Min Park, Ke Zheng, Ha Jeung Park, Veronica Soloveva, Dima Gharaibeh, Cary Retterer, Rouzbeh Zamani, Margaret L. Pitt, John Naughton, Yongjun Jiang, Hong Shang, Ramin M. Hakami, Binhua Ling, John A. T. Young, Sina Bavari, Xuehua Xu, Yangbo Feng, Yuntao Wu

**Affiliations:** a National Center for Biodefense and Infectious Diseases, George Mason University, Manassas, Virginia, USA; b Department of Laboratory Medicine, China Medical University, Shenyang, China; c Medicinal Chemistry, Translational Research Institute, The Scripps Research Institute, Jupiter, Florida, USA; d U.S. Army Medical Research Institute of Infectious Diseases, Frederick, Maryland, USA; e Henry M. Jackson Foundation, Bethesda, Maryland, USA; f Nomis Foundation Laboratories for Immunobiology and Microbial Pathogenesis, Salk Institute of Biological Studies, La Jolla, California, USA; g Tulane National Primate Research Center, Covington, Louisiana, USA; h Laboratory of Immunogenetics, National Institute of Allergy and Infectious Diseases, Rockville, Maryland, USA; i Reaction Biology Corporation, Malvern, Pennsylvania, USA; Ulm University Medical Center

**Keywords:** CXCR4, LIM domain kinase, Rift Valley fever virus, Venezuelan equine encephalitis virus, actin, cofilin, cytoskeleton, Ebola virus, herpes simplex virus, human immunodeficiency virus

## Abstract

A dynamic actin cytoskeleton is necessary for viral entry, intracellular migration, and virion release. For HIV-1 infection, during entry, the virus triggers early actin activity by hijacking chemokine coreceptor signaling, which activates a host dependency factor, cofilin, and its kinase, the LIM domain kinase (LIMK). Although knockdown of human LIM domain kinase 1 (LIMK1) with short hairpin RNA (shRNA) inhibits HIV infection, no specific small-molecule inhibitor of LIMK has been available. Here, we describe the design and discovery of novel classes of small-molecule inhibitors of LIMK for inhibiting HIV infection. We identified R10015 as a lead compound that blocks LIMK activity by binding to the ATP-binding pocket. R10015 specifically blocks viral DNA synthesis, nuclear migration, and virion release. In addition, R10015 inhibits multiple viruses, including Zaire ebolavirus (EBOV), Rift Valley fever virus (RVFV), Venezuelan equine encephalitis virus (VEEV), and herpes simplex virus 1 (HSV-1), suggesting that LIMK inhibitors could be developed as a new class of broad-spectrum antiviral drugs.

**IMPORTANCE** The actin cytoskeleton is a structure that gives the cell shape and the ability to migrate. Viruses frequently rely on actin dynamics for entry and intracellular migration. In cells, actin dynamics are regulated by kinases, such as the LIM domain kinase (LIMK), which regulates actin activity through phosphorylation of cofilin, an actin-depolymerizing factor. Recent studies have found that LIMK/cofilin are targeted by viruses such as HIV-1 for propelling viral intracellular migration. Although inhibiting LIMK1 expression blocks HIV-1 infection, no highly specific LIMK inhibitor is available. This study describes the design, medicinal synthesis, and discovery of small-molecule LIMK inhibitors for blocking HIV-1 and several other viruses and emphasizes the feasibility of developing LIMK inhibitors as broad-spectrum antiviral drugs.

## INTRODUCTION

Human immunodeficiency virus (HIV) infection causes CD4 T cell depletion and immunodeficiency. The virus can be effectively inhibited by antiretroviral drugs that target viral proteins, such as reverse transcriptase, protease, and integrase (1). However, the low fidelity of the viral reverse transcriptase promotes high levels of viral mutation, frequently leading to HIV drug resistance (2). HIV infection can also be inhibited by targeting host dependency factors (HDFs) (3–5). HDFs are cellular proteins that are functionally required for the completion of the viral life cycle. Viruses such as HIV interact with HDFs for essential functions, and thus, targeting HDFs can inhibit viral replication. A major advantage of developing HDF-based antiviral drugs is that it is difficult for viruses to generate drug resistance.

Recent studies have suggested that a dynamic actin cytoskeleton is necessary for viral entry, intracellular migration, and virion release (6–8). Viruses frequently devise various strategies to hijack cellular signaling pathways that regulate actin dynamics (6–8). During entry, HIV triggers early actin activity through binding of the viral envelope glycoprotein, gp120, to the chemokine coreceptor, CXCR4 (X4) or CCR5 (R5); this interaction activates heterotrimeric G proteins and downstream signaling pathways and actin modulators, such as cofilin and its kinase, the LIM domain kinase (LIMK) (through Rac1-PAK1/2-LIMK-cofilin) (8–13). Inhibition of LIMK1 activity and HIV-mediated actin dynamics has been shown to block HIV entry, nuclear migration, viral release, and cell-cell transmission, suggesting that LIMK1 is a host dependency factor necessary for HIV infection (11, 14; L. C. Zony and B. K. Chen, presented at the 2015 Meeting on Retroviruses, Cold Spring Harbor Laboratory, 18 to 23 March 2015, Cold Spring Harbor, NY, USA).

To inhibit HIV infection, LIMK1 has been stably knocked down (80 to 90%) by short hairpin RNA (shRNA) in human CD4 T cells, and this rendered T cells resistant to HIV infection (11); the LIMK1 knockdown cells permitted lower viral entry, viral DNA synthesis, and nuclear migration (11). In addition, it has been recently shown that LIMK is required for HIV-1 and Mason-Pfizer monkey virus (MPMV) particle release (14); small interfering RNA (siRNA) knockdown of LIMK1 prevents mature virions from leaving the plasma membrane. LIMK1 was also suggested to be involved in HIV-1 cell-cell transmission; disruption of LIMK1 greatly diminished HIV spread between cells (14; L. C. Zony and B. K. Chen, presented at the 2015 Meeting on Retroviruses, Cold Spring Harbor Laboratory, 18 to 23 March 2015, Cold Spring Harbor, NY, USA). For other viruses, LIMK1 was recently implicated in infection by influenza A virus (15), pseudorabies virus (16), and herpes simplex virus 1 (HSV-1) (17). During entry into neurons, HSV-1 triggers biphasic remodeling of the actin cytoskeleton through phosphorylation and dephosphorylation of cofilin, which is mediated through the HSV-1-induced epidermal growth factor receptor (EGFR)–phosphatidylinositol 3-kinase (PI3K)–Erk1/2–ROCK–LIMK1/2–cofilin signaling pathway (18). The pseudorabies virus also encodes a serine/threonine kinase, US3, that induces dramatic actin rearrangement by modulating cofilin. This US3-induced cofilin/actin activity facilitates viral spread (16). These recent studies suggested that targeting LIMK, the cofilin kinase, could inhibit the entry, release, and transmission of HIV and other viruses. However, for antiviral drug development, only a few small molecules have been shown to nonspecifically modulate LIMK activity (19–22), and none has demonstrated high selectivity. In this article, we describe the design, medicinal synthesis, and discovery of small-molecule LIMK inhibitors for blocking HIV-1 and several other viruses.

## RESULTS

### Medicinal chemistry and screening of LIMK inhibitors.

**FIG 1:**
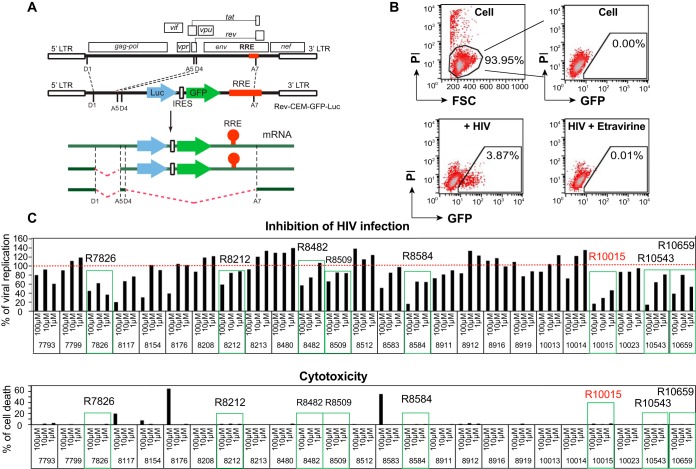
Screening LIMK inhibitors for anti-HIV activity. (A) Schematics of the Rev-dependent reporter construct and its transcripts in the HIV Rev-dependent indicator cell line Rev-CEM-GFP-Luc. The presence of Rev response element (RRE) in unspliced and singly spliced transcripts renders GFP/Luc expression Rev dependent. (B) Examples of HIV-dependent expression of GFP in Rev-CEM-GFP-Luc cells. Cells were not infected or infected with HIV(NL4-3) and treated with the reverse transcriptase inhibitor etravirine (100 nM). The cells were washed to remove the virus and the inhibitors and incubated for 48 h. HIV-dependent GFP expression was measured by flow cytometry. PI was added during flow cytometry to simultaneously measure cell viability. To exclude nonspecific cytotoxicity, only viable cells were used for GFP quantification. (C) Screening LIMK inhibitors with Rev-CEM-GFP-Luc cells. Cells were pretreated with LIMK inhibitors or DMSO for 1 h and then infected with HIV-1(NL4-3) for 3 h. The cells were washed to remove the virus and the inhibitors and incubated for 48 h. HIV-dependent GFP expression was measured by flow cytometry as described for panel B. PI was added during flow cytometry. The relative infection rates in drug-treated versus DMSO-treated cells (100%) were plotted using the relative percentage of GFP^+^ cells.

**FIG 2:**
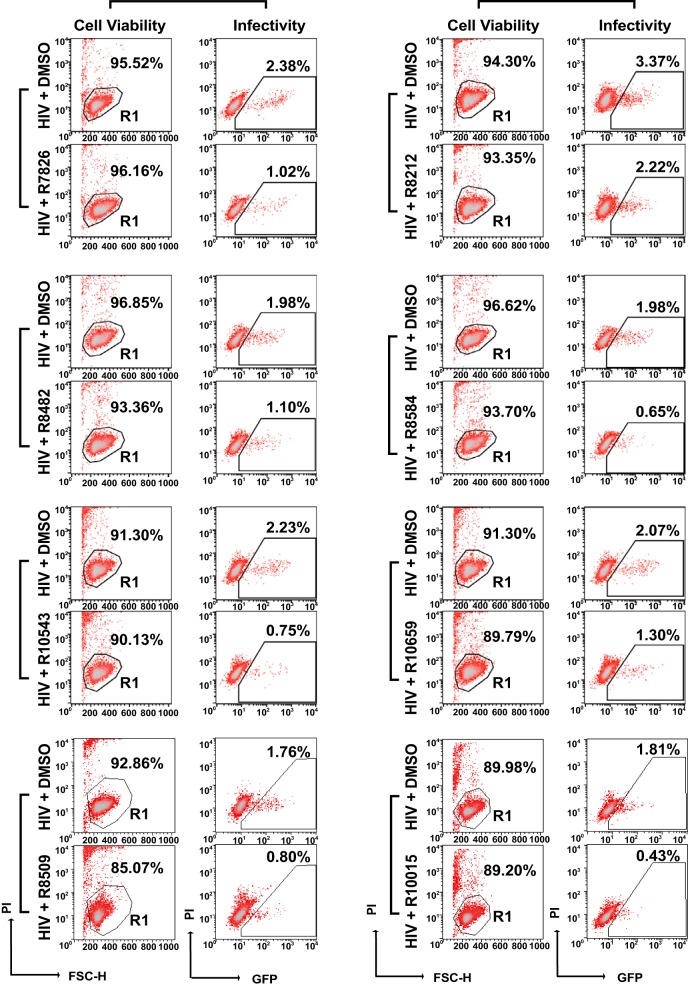
LIMK inhibitors discovered from anti-HIV screening. Rev-CEM-GFP-Luc HIV Rev-dependent indicator T cells were pretreated with LIMK inhibitors (100 μM) or DMSO for 1 h and then infected with HIV-1(NL4-3) for 3 h. The cells were washed to remove the virus and the inhibitors and incubated for 48 h. HIV-dependent GFP expression was measured by flow cytometry. PI was added during flow cytometry to simultaneously measure cell viability. Only viable cells (gated R1) were used for GFP quantification. The percentages of GFP^+^ cells are shown.

During our preliminary medicinal chemistry efforts, we designed and developed a series of small molecules that have the ability to inhibit LIMK in cell-free kinase assays. Given the requirement for LIMK in HIV infection (11, 14; L. C. Zony and B. K. Chen, presented at the 2015 Meeting on Retroviruses, Cold Spring Harbor Laboratory, 18 to 23 March 2015, Cold Spring Harbor, NY, USA), we performed further screening of these compounds for inhibiting HIV in live cells, using a newly constructed HIV Rev-dependent indicator cell line (23, 24), Rev-CEM-GFP-Luc. This Rev-dependent indicator cell expresses both green fluorescent protein (GFP) and luciferase (Luc) only in the presence of Rev from HIV infection (Fig. 1A and B) and has a high specificity that eliminates the nonspecificities associated with the commonly used long terminal repeat (LTR) promoter-driving reporter cells (23–31); in LTR-driving reporter cells (32–34), Tat- or HIV-independent reporter expression can be nonspecifically modulated by cellular inhibitors (e.g., HDAC inhibitors), cytokines, mitogens, or free viral proteins, such as gp120 (32, 35–40), diminishing the possibility of discovering antiviral drugs targeting HDFs. The high stringency of Rev-CEM-GFP-Luc cells permitted the screening of anti-HIV activity from LIMK inhibitors. As shown in Fig. 1C, we screened approximately 25 of these newly designed small molecules and identified eight lead compounds (R7826, R8212, R8482, R8509, R8584, R10015, R10543, and R10659) that blocked the X4 HIV infection at nontoxic dosages (Fig. 1C and 2).

**FIG 3:**
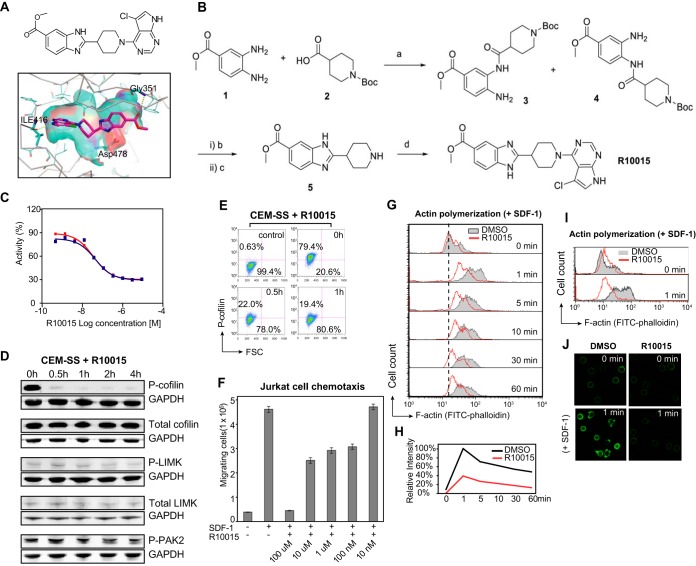
Chemical and biochemical characterization of R10015. (A) Chemical structure of R10015 and its docking into the crystal structure of LIMK1 (PDB accession no. 3S95, chain A). The binding motif of R10015 shows that it is a typical type I ATP-competitive kinase inhibitor. (B) R10015 synthesis. (a**)** EDC/HOBt/DIEA in DMF at room temperature for 16 h. (b) Acetic acid at 70°C for 4 h. (c) TFA (30%) in DCM at room temperature for 1 h. (d) 4,5-Dichloro-7H-pyrrolo[2,3-*d*]pyrimidine, DIEA, and isopropanol; 130°C; microwave for 3 h. (C) Ten-dose inhibition curve of R10015 against purified recombinant LIMK1 enzyme (IC_50_ = 38 ± 5 nM). (D) R10015 blocks cofilin serine 3 phosphorylation in human T cells. CEM-SS T cells were treated with R10015 (100 μM) for a time course, and the phosphorylation of cofilin, LIMK, and PAK2 was measured by Western blotting. GAPDH was used as a loading control. (E) Cofilin phosphorylation in R10015-treated (100 μM) CEM-SS T cells was also quantified by intracellular staining with an anti-p-cofilin antibody and analyzed by flow cytometry. The control was the background staining of cells in the absence of the anti-p-cofilin antibody. (F) R10015 inhibits Jurkat T cell chemotaxis in responding to SDF-1. Cells were treated with R10015 (100 μM) and added to the upper chamber of a 24-well transwell plate. The lower chamber was filled with SDF-1 (40 ng/ml). The plate was incubated at 37°C for 2 h, and the cells that migrated to the lower chamber were counted. FSC, forward scatter. The error bars indicate standard deviation. (G) R10015 inhibits chemotactic actin activity. R10015-treated (100 μM) resting CD4 T cells were exposed to SDF-1. Actin polymerization was measured by FITC-phalloidin staining. (H) The relative intensity of F-actin staining was also plotted. (I and J) Repeat of the experiment in panel H in another donor. Actin polymerization was quantified by flow cytometry (I) and confocal fluorescence microscopy (J).

Structurally, these lead LIMK inhibitors represent three chemotypes (41). Scaffold A is based on the 4-yl-piperidine- or piperazine-benzimidazole derivative; scaffold B is derived from phenyl benzimidazole analogs, and scaffold C is based on the bis-aryl urea moiety. All three scaffolds share a substituted or unsubstituted 7H-pyrrolo[2,3-d]pyrimidine moiety (Table 1). We also performed a molecular docking study using R10015 (Fig. 3A) and the published crystal structure of LIMK1 (Protein Data Bank [PDB] accession no. 3S95). The result demonstrated that this pyrrolopyrimidine moiety functions as an ATP site hinge binding group bound to the backbone of the hinge residue Ile416 with H-bonding interactions from the NH/N of pyrrolopyrimidine (Fig. 3A). Our docking study suggests that all the compounds from these three scaffolds are type I ATP-competitive kinase inhibitors. In addition to the hinge interactions, the terminal aromatic moiety of these LIMK inhibitors is bound to a pocket under the P loop with strong hydrophobic interactions (Fig. 3A). An additional H-bonding interaction(s), which could contribute to the high affinity of these LIMK inhibitors, might also be present in this binding motif, depending on the functional substitution(s). For example, for R10015, there is also a potential H-bonding interaction between the ester carbonyl group and the backbone amide of residue Gly351 (Fig. 3A and B).

**TABLE 1:**
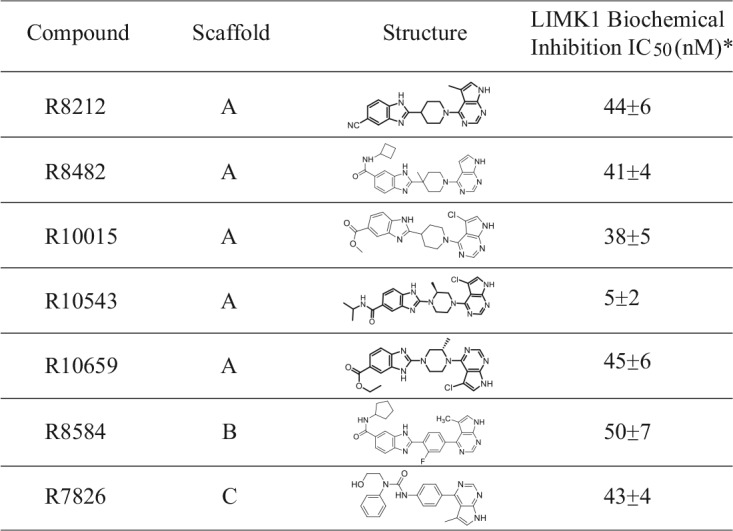
LIMK inhibitors identified from anti-HIV screening^*a*^

a *, IC_50_s are from ≥2 *in vitro* kinase assays using purified LIMK1.

### Molecular and cellular characterization of the LIMK inhibitor R10015.

We selected R10015 from the potential LIMK inhibitors for further detailed mechanistic studies. The synthesis, purification, and biochemical characterization of R10015 are described in Materials and Methods below and shown in Fig. 3. We performed a cell-free assay to measure R10015 inhibition of the LIM domain kinase using purified human LIMK1 and its substrate, cofilin. The biochemical half-maximal inhibition concentration (IC_50_) of R10015 against purified LIMK1 was determined to be approximately 38 nM (Fig. 3C). We also performed profiling of R10015 against a panel of 62 kinases (Table 2). The results demonstrated that R10015 has good selectivity, with off-target inhibition of only LRRK2 and p70S6K (≥90% inhibition at 1 μM R10015) and moderate inhibition of protein kinase A (PKA) (∼76%), ROCK2 (∼70%), and FLT3 (∼68%).

**TABLE 2:**
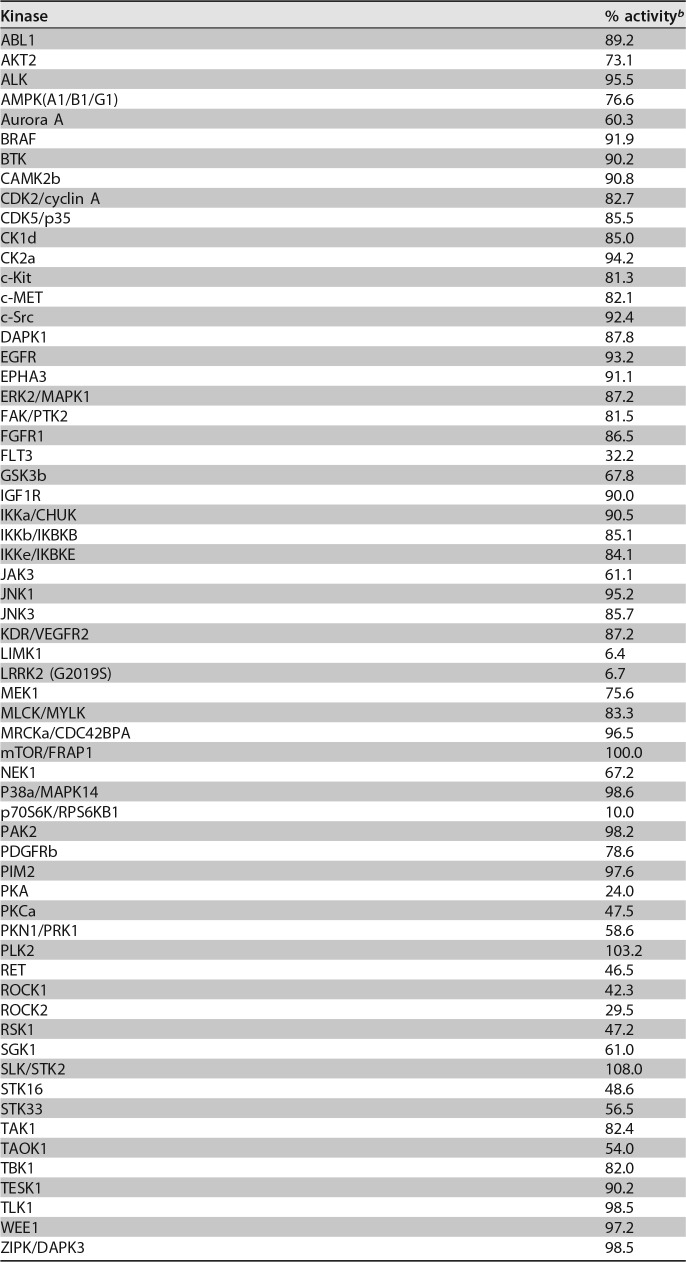
Profile of R10015 (1 μM) against 62 kinases^*a*^

a Profiling was carried out at Reaction Biology Corporation following a protocol described on its website.

b The activity data are averages of two determinations (DMSO control, 100% activity). The ATP concentration used was 10 μM; staurosporine was used as the control for assay validation.

To further determine the specificity of R10015 in human CD4 T cells, we treated CEM-SS T cells with R10015 for a time course. Similar to the cell-free assay, we used LIMK-mediated cofilin phosphorylation as the readout. Following R10015 treatment, the cells were lysed, and cofilin phosphorylation was measured by Western blotting. As shown in Fig. 3D, we detected drastic inhibition of cofilin serine 3 phosphorylation, confirming that R10015 blocks the LIMK activity in cells. The decrease of phospho-cofilin (p-cofilin) did not result from possible inhibition of cofilin protein synthesis. When total protein levels were quantified, R10015 did not cause a decrease in the total amount of cofilin (Fig. 3D). The total protein levels of LIMK itself were also not decreased (Fig. 3D). To further confirm that the inhibition of cofilin phosphorylation occurs directly through blocking LIM kinase, we examined upstream signaling molecules that regulate LIMK activity. We found that R10015 did not inhibit the threonine 508/505 phosphorylation of LIMK1/2 itself by the upstream kinase PAK2, whose activity is regulated by upstream Rho family GTPases, such as Rac, cdc42, and Rho (11, 42) (Fig. 3D). The phosphorylation of PAK2 was also not affected by R10015 (Fig. 3D). These results strongly suggest that the inhibition of cofilin serine 3 phosphorylation by R10015 came from the direct inhibition of the activity of LIMK; the LIMK upstream kinases and GTPases are not the targets of R10015. These cell-based studies confirmed the high selectivity of R10015 in blocking the LIM kinase activity in human CD4 T cells.

The Western blot quantification of R10015 on cofilin phosphorylation (Fig. 3D) represents the drug effect on cells as a whole population. We further quantified cofilin phosphorylation in individual T cells by intracellular staining and flow cytometry. Cells were treated with R10015 for a time course, permeabilized, and stained with a rabbit anti-human p-cofilin antibody, followed by staining with Alexa Fluor 647-labeled chicken anti-rabbit antibodies. Stained cells were analyzed by flow cytometry. As shown in Fig. 3E, in cells not treated with R10015 (0 h), approximately 80% of the cells stained relatively high on p-cofilin. Following R10015 treatment for 1 h, 80% of the cells stained low on p-cofilin. These results confirm that R10015 inhibits cofilin phosphorylation in a majority of T cells.

We further tested R10015 for inhibition of stromal cell-derived factor 1 (SDF-1)-mediated chemotaxis and actin dynamics, which are known to be regulated by LIMK (43). Human Jurkat T cells were treated with different doses of R10015 and then placed into the upper chamber of a transwell plate. SDF-1 was added to the lower chamber. The migration of the cells to the lower chamber was enumerated. As shown in Fig. 3F, we observed a dose-dependent inhibition of Jurkat T cell chemotaxis, and the IC_50_ was around 10 μM, which is higher than the IC_50_ (38 nM) observed in the *in vitro* LIMK assay (Fig. 3C). This difference in R10015 potency between the *in vitro* assay and that in live cells likely resulted from nonoptimal properties of R10015 for intracellular delivery, which may require further medicinal chemistry optimization. To further confirm that the inhibition of SDF-1-mediated chemotaxis is directly related to R10015-mediated inhibition of actin dynamics, we measured actin polymerization following SDF-1 and R10015 treatment. For measuring actin polymerization, human blood resting CD4 T cells were treated with R10015 and SDF-1 for a time course. The cells were permeabilized, stained with fluorescein isothiocyanate (FITC)-phalloidin for F-actin, and analyzed by flow cytometry. Consistently, pretreatment of resting CD4 T cells with R10015 markedly depressed SDF-1-mediated actin polymerization (Fig. 3G and H), confirming that R10015 blocks LIMK-regulated actin dynamics. Confocal microscopy observation of R10015-treated T cells revealed that the drug dramatically blocked actin polymerization and actin capping following SDF-1 stimulation (Fig. 3I and J).

### Mechanistic study of R10015 for blocking HIV infection.

**FIG 4:**
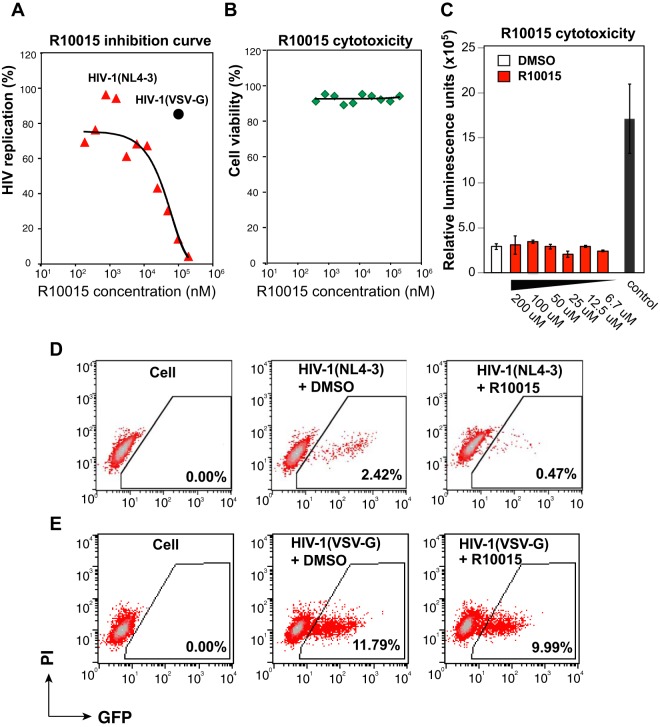
R10015 inhibition of HIV infection of human T cells. (A) Rev-CEM-GFP-Luc cells were pretreated with different doses of R10015 for 1 h and then infected with HIV-1(NL4-3) for 3 h. The cells were washed to remove the virus and the inhibitor and incubated for 48 h. HIV-dependent luciferase expression was quantified, and the IC_50_ of R10015 was calculated (red triangles). For comparison, cells were also treated with 100 μM R10015 and infected with a VSV G-pseudotyped HIV for 3 h (black circle). Following infection, the cells were washed to remove the virus and R10015 and incubated for 48 h. (B) The cytotoxicity of R10015 was simultaneously measured by PI staining and flow cytometry. (C) The cytotoxicity of R10015 was also measured using a luciferase-based multiplex cytotoxicity assay, as described in Materials and Methods. For cytotoxicity control, 1% saponin was added to the cells to induce cytotoxicity (control). The error bars indicate standard deviations. (D and E) Flow cytometry results demonstrating that R10015 inhibited HIV-1(NL4-3) but minimally inhibited HIV-1(VSV G) when the cells were briefly treated with R10015 (100 μM) early during viral infection.

Previous shRNA/siRNA LIMK knockdown studies have demonstrated that LIMK is involved in viral entry, DNA synthesis, nuclear migration, and viral budding (11, 14). We first quantified R10015 inhibition of the early steps of HIV infection of CD4 T cells, in which cells were exposed to R10015 only briefly during viral infection; R10015 was removed from the infection culture following infection. As shown in Fig. 4, Rev-CEM-GFP-Luc cells were pretreated with different doses of R10015 for 1 h, infected with HIV for 3 h, washed to remove the input virus and R10015, and then cultured in the absence of R10015 for 48 h. HIV-dependent luciferase expression was quantified. For inhibition of HIV, R10015 showed an IC_50_ of 14.9 μM (Fig. 4A), which is close to the IC_50_ (10 μM) for inhibition of SDF-1-mediated chemotaxis (Fig. 3F). The drug had no detectable cytotoxicity for this short period of treatment (4 h) at all the concentrations tested (up to 200 μM) (Fig. 4B and C). In addition, R10015 minimally inhibited the early steps of vesicular stomatitis virus (VSV) G-pseudotyped HIV, even at 100 μM (Fig. 4A and E), confirming that the inhibition of HIV did not result from nonspecific cytotoxicity and is indeed specific to viral processes related to HIV gp120-mediated fusion and entry (44); HIV gp120-mediated infection of human CD4 T cells has been known to require cortical actin dynamics (8). On the other hand, VSV G-mediated endocytosis bypasses cortical actin (45) and thus is less susceptible to R10015. This differential susceptibility of gp120- versus VSV G-pseudotyped virus to R10015 is in agreement with the hypothesis that R10015 inhibits HIV through direct blockage of the LIMK-regulated actin dynamics.

**FIG 5:**
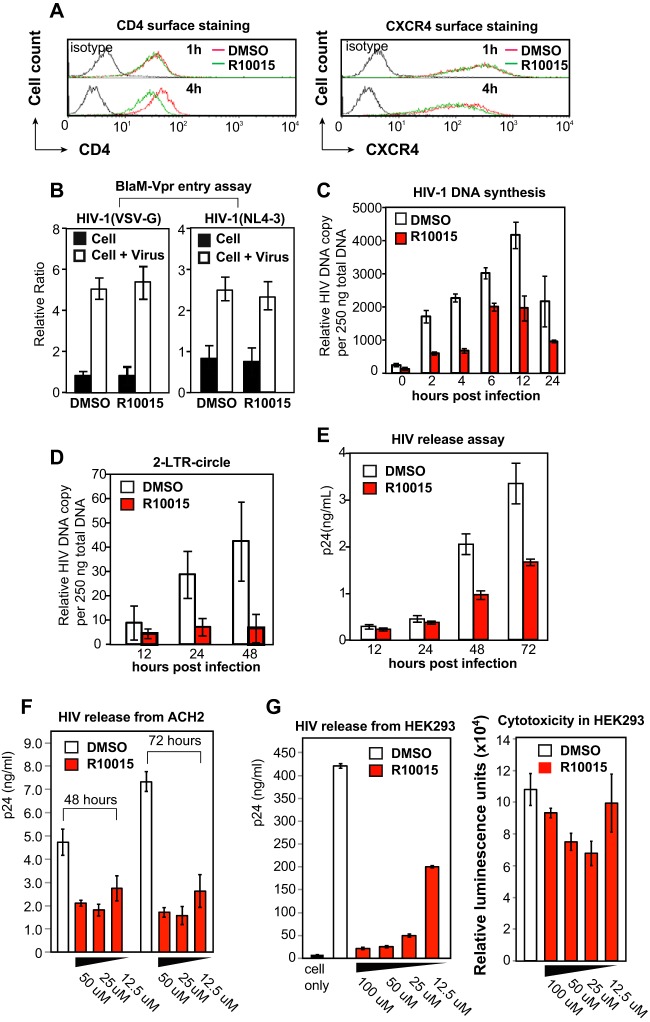
R10015 inhibits HIV-1 DNA synthesis, nuclear migration, and virion release. (A) Effects of R10015 on surface CD4 and CXCR4 expression. CEM-SS T cells were treated with R10015 (100 μM) and then stained for surface CD4 or CXCR4. (B) R10015 did not inhibit viral entry. CEM-SS T cells were treated with R10015 (100 μM) and then infected with BlaM-Vpr-tagged HIV-1(NL4-3) or HIV-1(VSV G) to measure viral entry. (C) R10015 inhibits viral DNA synthesis. CEM-SS T cells were treated with R10015 (100 μM) for 1 h and then infected with a single-cycle HIV-1(Env) for 2 h in the presence of R10015. Following infection, the cells were washed to remove HIV-1 and R10015. Viral DNA synthesis was measured by real-time PCR. (D) R10015 inhibits 2-LTR circle DNA formation. Cells were similarly treated with R10015 and infected. 2-LTR circles were quantified by real-time PCR. (E) R10015 inhibits virion release. Cells were infected with HIV-1(Env) for 2 h, washed, incubated for 12 h, and then treated with R10015. Virion release was quantified by measuring p24 in the supernatant. (F) R10015 inhibits virion release from chronically infected ACH2 cells. ACH2 cells were washed 3 times and then treated with R10015 at various dosages. The cells were cultured for 2 to 3 days. Culture supernatants were harvested and analyzed for HIV-1 p24 by ELISA. DMSO was used as a control. (G) R10015 inhibits virion release from DNA-transfected HEK293 cells. The cells were transfected with plasmid pHIV-1(NL4-3) and then treated with different concentrations of R10015 for 48 h. Culture supernatants were harvested and analyzed for HIV-1 p24 by ELISA. DMSO was used as a control. The cytotoxicity of R10015 in HEK293 cells was also measured using a luciferase-based multiplex cytotoxicity assay, as described in Materials and Methods. The error bars indicate standard deviations.

To further elucidate the anti-HIV mechanism, we pretreated human CEM-SS T cells with R10015 for 1 h, and the effects of R10015 on the surface expression of the HIV receptors, both CD4 and CXCR4, were examined. This brief treatment did not alter the surface expression of CD4 or CXCR4; prolonged treatment (4 h), however, slightly decreased the surface density of CD4 (Fig. 5A) (11). We further examined the effect of R10015 on viral entry using two HIV entry assays, the Vpr-β-lactamase- and Nef-luciferase-based assays (46, 47). R10015 did not significantly inhibit viral entry, as measured by infection with HIV-1(Vpr-β-lactamase) (46) (Fig. 5B) or HIV-1(Nef-luciferase) virion particles (reference 47 and data not shown). We then followed viral postentry steps, such as viral DNA synthesis and nuclear migration. For these stepwise mappings, we used a single-cycle virus, HIV-1(Env), which is pseudotyped with HIV-1 gp160 (45). CEM-SS T cells were pretreated with R10015 for 1 h and then infected with HIV-1(Env) for 2 h in the presence of R10015. Following infection, the cells were washed to remove HIV-1 and R10015 and cultured for 24 to 48 h. The late products of HIV reverse transcription were quantified by real-time PCR. As shown in Fig. 5C, R10015 inhibited viral late DNA synthesis at all time points. We also measured HIV nuclear migration using the synthesis of HIV circular DNA containing 2 copies of the viral long terminal repeat (2-LTR circles) as a surrogate. R10015 also inhibited viral nuclear migration, as measured by viral 2-LTR circles (Fig. 5D).

Recently, LIMKs have been suggested to be involved in retroviral budding and release (14); siRNA knockdown of LIMK1 prevents mature virions from leaving the plasma membrane. We further tested the effects of R10015 on the late stage of HIV infection. CEM-SS T cells were infected with HIV-1(Env) for 2 h, washed, incubated for 12 h, and then treated with R10015. Virion release from 12 to 72 h postinfection was quantified by measuring p24 release into the supernatant. As shown in Fig. 5E, R10015 also inhibited p24 release when applied at a later stage of HIV infection, confirming previous findings on LIMK in HIV budding and release (14). As a complementary approach, we also treated a chronically HIV-infected T cell line, ACH-2, with R10015, and observed similar inhibition of virion release (Fig. 5F). Furthermore, R10015 also inhibited virion release from HIV DNA-transfected HEK293 cells (Fig. 5G); the use of DNA transfection to assemble virion particles abrogates the requirements for viral early steps, such as entry, reverse transcription, and integration. Thus, the inhibition of virion release by R10015 is associated with direct blocking of viral late steps rather than early steps. Together, our results demonstrate that R10015 inhibited viral DNA synthesis, nuclear migration, and virion release, phenotypes consistent with those observed in shRNA/siRNA LIMK1 knockdown cells (11, 14).

**FIG 6:**
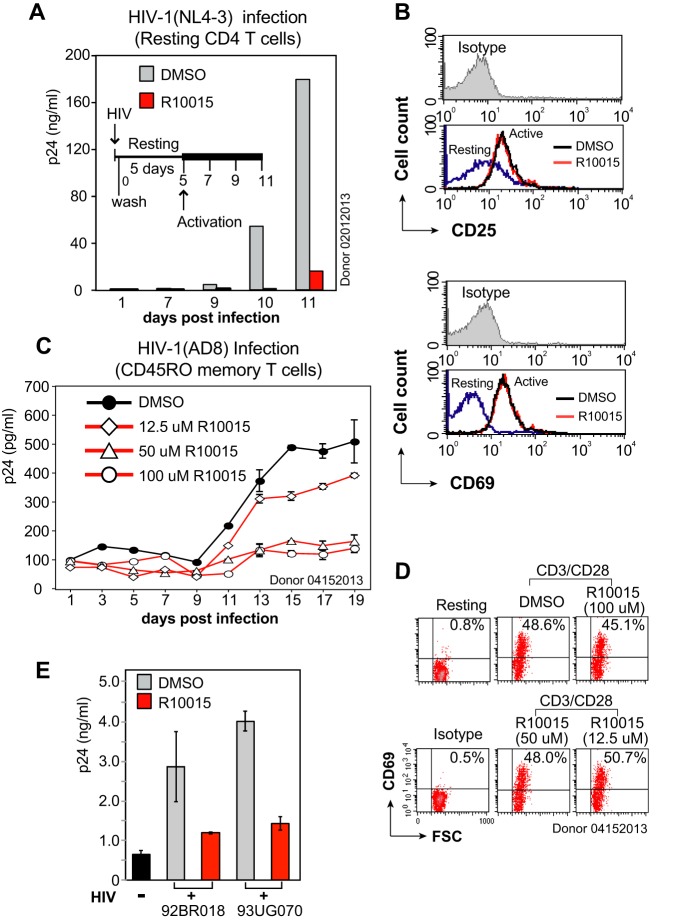
R10015 inhibits R5 and X4 HIV-1 latent infection of resting CD4 T cells and primary isolate infection of PBMC. (A) R10015 inhibits HIV latent infection of resting CD4^+^ T cells. Cells were treated with R10015 (100 μM) or DMSO for 1 h and infected with HIV-1(NL4-3) for 2 h. The virus and the drug were washed away, and the cells were cultured for 5 days in the absence of R10015 and then activated with CD3/CD28 beads. Viral p24 release was measured. (B) CD25 and CD69 surface staining demonstrates that R10015 did not inhibit T cell activation with this short period of drug treatment. (C) R10015 inhibits R5 HIV-1 latent infection of CD45RO^+^ memory CD4 T cells. Resting memory CD4 T cells were similarly treated with R10015, infected with HIV-1(AD8), washed, incubated for 5 days without stimulation, and then activated with CD3/CD28 beads. (D) CD69 surface staining was performed for control of R10015 effects on T cell activation. (E) R10015 inhibits HIV-1 primary isolate infection of PBMC. PBMC were cultured for 1 day and then treated with 100 μM R10015 for 1 h. The cells were infected with HIV 92/BR/018 (Brazil) or HIV 93UG070 (Uganda) for 3 h, washed to remove the viruses and R10015, and cultured for 3 days. The supernatant was analyzed for HIV-1 p24 by ELISA. DMSO was used as a control. The error bars indicate standard deviations.

During HIV transmission, early viruses utilize CCR5 and mainly infect memory CD4 T cells and macrophages, whereas late viruses also utilize CXCR4 (48–50). We tested the ability of R10015 to inhibit R5 and X4 latent infection of blood memory and resting CD4 T cells (8, 51). In our viral latent infection system, total resting CD4 T cells or the CD45RO^+^ RA^−^ memory CD4 T cells were purified from the peripheral blood, cultured overnight without stimulation, and then treated with R10015 for 1 h. Resting T cells were infected with HIV-1(NL4-3) for 2 h, washed, cultured for 5 days in the absence of R10015, and then activated with CD3/CD28 beads to stimulate viral replication. Viral p24 release was measured. As shown in Fig. 6, we observed that R10015 blocked X4 HIV-1 latent infection of resting CD4 T cells (Fig. 6A); we also observed R10015 dose-dependent inhibition of R5 HIV latent infection of resting memory CD4 T cells (Fig. 6C). The inhibition of HIV latent infection of resting T cells did not result from possible drug cytotoxicity, because the short drug treatment did not inhibit T cell function, as judged by the surface expression of CD25 or CD69 following T cell activation (Fig. 6B and D). These results confirmed that the LIMK inhibitor is effective in blocking both X4 and R5 viral infection of primary target cells. We further tested the ability of R10015 to inhibit infection by primary HIV isolates. As shown in Fig. 6E, HIV 92/BR/018 (Brazil) and HIV 93UG070 (Uganda) were used to infect peripheral blood mononuclear cells (PBMC) in the presence of R10015. We observed inhibition of HIV replication by R10015.

### Inhibition of multiple viruses by R10015.

**FIG 7:**
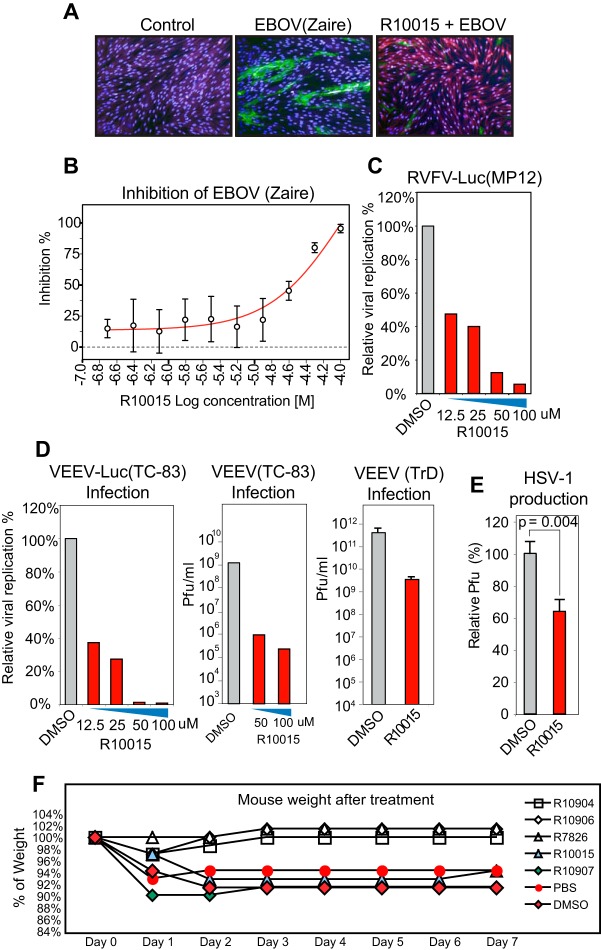
R10015 inhibition of EBOV, VEEV, RVFV, and HSV-1. (A and B) Inhibition of EBOV by R10015. (A) HFF-1 cells were treated with R10015 (50 μM) for 2 h and infected with EBOV (Zaire) (MOI, 2.5) for 48 h in the presence of R10015. The cells were fixed and stained for the EBOV GP protein with an Alexa 488-labeled antibody (green) or with Hoechst (blue nuclei) for confocal imaging. (B) The relative GP protein staining was converted to percent inhibition using the infected, non-drug-treated cells as the control. (C) Inhibition of RVFV by R10015. Vero cells were similarly treated with R10015, infected with RVFV-Luc (MP12) (MOI, 0.1), and analyzed by luciferase assay. (D) Inhibition of VEEV by R10015. Vero cells were similarly treated with R10015 and infected with VEEV-Luc(TC-83), VEEV(TC-83), or VEEV(TrD) (MOI, 0.1). The viral supernatants were collected at 24 h and analyzed by luciferase or plaque assay. (E) Inhibition of HSV-1 by R10015. Vero cells were treated with R10015 (100 μM) for 2 h, infected with HSV-1, washed, and cultured in the absence of R10015. Viral plaques were stained and quantified. No drug toxicity was observed up to 100 μM R10015. (F) C3H/HeN mice were treated daily with LIMK inhibitors for 7 days. R10904, R10906, R10907, and R7826 were delivered via oral gavage at 20 mg/kg. R10015 was delivered by intraperitoneal injection at 10 mg/kg. DMSO-treated and PBS-treated mice were used as controls. The animals were weighed daily and observed for signs of stress. The error bars indicate standard deviations.

Given that the requirement for actin dynamics is shared among viruses, we tested R10015 for its ability to inhibit several other viruses, including Zaire ebolavirus (EBOV), Rift Valley fever virus (RVFV), Venezuelan equine encephalitis virus (VEEV), and herpes simplex virus 1 (HSV-1). Ebola virus is a negative-sense, single-stranded RNA virus of the family Filoviridae. EBOV acquires an envelope from the host cell membrane during virion budding. The cellular entry of EBOV has been suggested to occur through a macropinocytosis-like mechanism that involves localized polymerization of the actin filaments (52). To test the possible effects of R10015, we pretreated human HFF-1 cells with R10015 for 2 h and then infected them with EBOV (Zaire) for 48 h in the continuous presence of R10015. To quantify viral replication, the cells were fixed and stained for the EBOV glycoprotein with an Alexa 488-labeled antibody and then analyzed by confocal imaging. We observed dose-dependent inhibition of EBOV (Fig. 7A and B). This inhibition is likely a combined effect of R10015 on viral early and late processes, such as intracellular migration and virion release.

We further tested the effects of R10015 on another negative-sense, single-stranded RNA virus, RVFV, of the bunyavirus family. RVFV is a cytoplasmic virus, and the cellular entry of RVFV is predicted to occur via a class II fusion mechanism that becomes activated by low pH following endocytosis of the virus (53). Given that R10015 is less effective in inhibiting endocytotic entry mediated by VSV G (Fig. 4C), we treated cells with R10015 for the entire course of viral replication to block both early and late steps, such as viral assembly. We used a luciferase-tagged virus, RVFV-Luc (MP12), to measure viral replication. As shown in Fig. 7C, we observed dosage-dependent inhibition of RVFV with an IC_50_ at approximately 12.5 μM. We also tested a single-stranded, positive-sense RNA virus, VEEV, a member of the alphaviruses. The cellular entry of VEEV is also believed to be dependent on clathrin-mediated endocytosis (54). To block VEEV replication, we also treated cells with R10015 for the entire course of viral replication. We used a luciferase-tagged virus, VEEV-Luc (TC-83), and the attenuated VEEV (TC-83) (biosafety level 2 [BSL-2]) and observed dosage-dependent inhibition in both luciferase and plaque assays with an IC_50_ of approximately 5 μM (Fig. 7D). We confirmed these results with the infectious VEEV (TrD) strain (BSL3), and observed 2 log units of inhibition of VEEV (TrD) with R10015 (Fig. 7D).

Recently, studies have suggested that LIMK is also involved in HSV-1 infection of neurons (18). During viral entry, HSV-1 triggers biphasic remodeling of the actin cytoskeleton through phosphorylation and dephosphorylation of cofilin, which is mediated through the LIMK-cofilin signaling pathway (18). To confirm the involvement of LIMK in the early infection steps of HSV-1 infection, we pretreated Vero cells with R10015 for 2 h and then infected them with HSV-1 for 2 h. Following infection, both HSV-1 and R10015 were washed away, and the cells were cultured in the absence of the drug. As shown in Fig. 7E, we observed inhibition of HSV-1 by R10015, even with this brief treatment, in agreement with a previous report that LIMK is involved in HSV-1 early infection steps. Together, our results demonstrate the broad antiviral activity of R10015.

To further test the feasibility of developing LIMK inhibitors for *in vivo* use, we used five different LIMK inhibitors to treat mice daily for 7 days (Fig. 7F). R10904, R10906, R10907, or R7826 was delivered via oral gavage at 20 mg/kg of body weight. Phosphate-buffered saline (PBS) was used as a control. R10015 (dissolved in dimethyl sulfoxide [DMSO]) was delivered via intraperitoneal (i.p.) injection at 10 mg/kg. DMSO was used as a control. The mice were weighed daily and monitored for morbidity and mortality, including lethargy and ruffled fur. None of the LIMK inhibitors displayed any indication of toxicity. In fact, mice treated with R10904, R10906, and R7826 gained weight over the course of a week compared to the PBS control. These results suggest the possibility of short-term use of LIMK inhibitors to block viral infections.

## DISCUSSION

In this article, we described the design, medicinal synthesis, and discovery of LIMK inhibitors for blocking HIV-1, EBOV, and other viruses. Our study is the first proof-of-concept study for developing LIMK inhibitors to block viruses. Our results emphasize the feasibility of using LIMK inhibitors as anti-HIV and broad-spectrum antiviral drugs. The rational design and development of LIMK inhibitors as antiviral drugs are largely based on recent studies on the role of LIMK/cofilin in HIV-1 infection (8, 11, 14). It has been shown that HIV requires LIMK1/cofilin activity for entry, intracellular migration, cell-cell transmission, and virion budding (8, 11, 14). Given that the requirement for actin dynamics is shared among viruses, these LIMK inhibitors are capable of inhibiting multiple viruses.

As a proof-of-concept study, our current lead compounds, particularly R10015, have low molecular weight (<450) and are amenable to further medicinal chemistry optimization to achieve higher solubility, cell potency, and appropriate drug metabolism and pharmacokinetic properties (DMPK). When profiled against a panel of 62 kinases *in vitro* (Table 2), R10015 demonstrated reasonably good selectivity (IC_50_, 38 nM for LIMK1), with off-target inhibition of only LRRK2 and p70S6K (≥90% inhibition at 1 μM) and moderate inhibition of PKA (∼76% inhibition at 1 μM), ROCK2 (∼70% inhibition at 1 μM), and FLT3 (∼68% inhibition at 1 μM). Nevertheless, there is still a possibility that the inhibition of HIV infection by R10015 may result from inhibition of multiple proteins. However, previous siRNA knockdown studies have demonstrated that the knockdown of LRRK2 or P70S6K did not inhibit HIV infection; the knockdown of FLT3, PKA, or ROCK2 also had no significant impact on HIV replication (55–57), whereas shRNA or siRNA knockdown of LIMK has been shown to inhibit HIV (11, 14; L. C. Zony and B. K. Chen, presented at the 2015 Meeting on Retroviruses, Cold Spring Harbor Laboratory, 18 to 23 March 2015, Cold Spring Harbor, NY, USA). These previous studies suggest that R10015-mediated inhibition of HIV infection is a direct result of inhibiting LIMK. To further ensure that R10015 blocks HIV by inhibiting LIMK, we performed a series of experiments demonstrating that (i) R10015 strongly blocked cofilin phosphorylation, whereas the other upstream kinases were largely unaffected, demonstrating its good selectivity (Fig. 3D); (ii) R10015 effectively blocked actin polymerization and T cell chemotaxis (Fig. 3F and G), as these are the expected properties of LIMK inhibitors; and (iii) most importantly, R10015 selectively blocked wild-type HIV infection, but not VSV G-pseudotyped HIV infection, when used early during viral entry (Fig. 4). This strongly suggests that R10015-mediated viral inhibition does not result from nonspecific cytotoxicity but is related to the inhibition of specific processes, such as actin dynamics, that are involved in HIV entry; it has been well documented that HIV gp120-mediated fusion and entry require cortical actin activities (8), while VSV G-mediated endocytotic entry is less dependent on actin dynamics (45). Furthermore, our detailed stepwise molecular mapping confirmed that R10015-mediated inhibition of viral nuclear entry (Fig. 5D) is similar to that observed in shRNA LIMK knockdown cells (11).

Given the critical role of LIMK in regulating cell migration, chemotaxis, and T cell activation, long-term suppression of LIMK may inhibit immune responses. Nevertheless, the possibility of developing future clinical LIMK inhibitors is suggested by genetic studies in humans and mice. In the human genome, the LIMK1 gene is located in a region on human chromosome 7 (7q11.23) and is haplodeleted, along with 24 other genes, in adults living with Williams syndrome (WS), a rare neurodevelopmental genetic disorder associated with mild mental retardation (58–60). The deletion of elastin (ELN) has been explicitly linked to most of the cardiovascular problems in WS (61). However, the neurodevelopmental genotype-phenotype correlation is still uncertain and may be related to the hemizygosity of WBSCR11, CYLN2, GTF2I, NCF1, and perhaps LIMK1 (60). Specifically, LIMK1 knockout in mice has been linked to alterations in spine morphology and synaptic function. The knockout mice also showed heightened locomotor activity and altered fear responses and spatial learning (62). Nevertheless, LIMK1-null mice and human adults with WS do not have the severe multiple developmental disorders normally seen in other developmental genetic diseases, suggesting that blocking LIMK1 is not fatal and that short-term or localized LIMK inhibition is likely tolerable in adults. Therefore, LIMK is considered a valuable target for treating various human diseases, such as metastatic cancer, Alzheimer's disease, and drug addiction (63–65). These previous studies are also consistent with our results showing that the LIMK inhibitors are generally nontoxic in our cell-based assays and in mice (Fig. 7F), demonstrating the feasibility of developing highly specific LIMK inhibitors for short-term use to inhibit HIV and other viruses. Given the lack of an effective HIV vaccine, these novel inhibitors may prove to be valuable alternatives for preventing HIV sexual transmission; these LIMK inhibitors inhibit viral reverse transcription, nuclear migration, and release and are expected to be effective against a broad spectrum of HIV strains because of the highly conserved nature of viral dependency on actin dynamics for infection. In addition, these LIMK inhibitors have anti-inflammatory properties and can inhibit the migration of infected immune cells for HIV cell-cell transmission (Fig. 3F) (14). Thus, these LIMK inhibitors are ideal candidates, as potential topical microbicides, for preexposure prophylaxis that may complement the current antiretroviral drugs. Finally, our results also emphasize the feasibility of developing LIMK inhibitors as a new class of broad-spectrum antiviral drugs for urgent treatment of exposure to viral agents.

## MATERIALS AND METHODS

### Approvals from IRB and IACUC.

Peripheral blood was drawn from HIV-negative donors. All protocols involving human subjects were reviewed and approved by the George Mason University (GMU) institutional review board (IRB). Mouse experiments were carried out in animal BSL-2 facilities in accordance with the National Research Council's Guide for the Care and Use of Laboratory Animals and under GMU IACUC protocol number 0211.

### Synthesis of R10015.

Commercially available reagents and anhydrous solvents were used without further purification unless otherwise specified. Thin-layer chromatography (TLC) analyses were performed with precoated silica gel 60 F254 plates. Mass spectra were recorded by liquid chromatography (LC)-mass spectrometry (MS) with a Finnigan LCQ Advantage Max spectrometer from Thermo Electron. Flash chromatography was performed on prepacked silica gel columns (230 to 400 mesh; 40 to 63 μm) by CombiFlash with ethyl acetate (EtOAc)-hexane or methanol (MeOH)-dichloromethane (DCM) as the eluent. Preparatory high-performance liquid chromatography (HPLC) was performed on a SunFire C_18_ OBD column, 10 μm (30 by 250 mm), with CH_3_CN plus 50% MeOH-H_2_O plus 0.1% trifluoroacetic acid (TFA) as the eluent to purify the targeted compounds. Analytic HPLC was performed on an Agilent Technologies 1200 series HPLC with CH_3_CN (solvent B)-H_2_O plus 0.9% CH_3_CN plus 0.1% TFA (solvent A) as the eluent; the targeted products were detected by UV in the detection range of 215 to 310 nm. Nuclear magnetic resonance (NMR) spectra were recorded with a Bruker 400-MHz spectrometer at ambient temperature, with the residual solvent peaks as internal standards. The line positions of multiplets are given in parts per million (δ), and the coupling constants (*J*) are given in hertz. The high-resolution mass spectrum (HRMS) (electrospray ionization) experiments were performed with a Thermo Finnigan Orbitrap mass analyzer. Data were acquired in the positive ion mode at a resolving power of 100,000 at *m/z* 400. Calibration was performed with an external calibration mixture immediately prior to analysis.

### General synthetic procedures.

The scheme and synthetic procedures described below are for the inhibitor R10015. The syntheses of the other LIMK inhibitors listed in Table 1 followed similar protocols. As shown in Fig. 3B, 1-ethyl-3-(3-dimethylaminopropyl) carbodiimide hydrochloride (EDC) (1.2 equivalent) was added to a stirring mixture of 1 (1 equivalent), 2 (1.05 equivalent), 1-hydroxybenzotriazole (HOBt) (1 equivalent), and diisopropylethylamine (DIEA) (3 equivalent) in dimethylformamide (DMF). Stirring was continued at room temperature overnight, after which LC-MS indicated a complete reaction. The solvent was removed *in vacuo* to residue, which was suspended in EtOAc. The suspension was washed with brine and saturated NaHCO_3_, dried over anhydrous Na_2_SO_4_, and evaporated under reduced pressure to give a mixture of crude amide products 3 and 4. Without further purification, the mixture was suspended in acetic acid and heated at 70°C for 4 h for ring closure to give the Boc-protected 4-yl-piperidinobenzimidazole, which was purified by flash chromatography. The Boc protection was then removed with 30% TFA in DCM to yield 5 as an oil. Finally, a mixture of 5 and 4,5-dichloro-7H-pyrrolo[2,3-*d*]pyrimidine in a small amount of isopropanol was heated at 130°C under microwave conditions for 3 h to produce the LIMK inhibitor R10015, which was purified by reverse-phase HPLC to a purity of >95% based on analytical HPLC analysis (UV detection at 254 nm).

### Methyl 2-(1-(5-chloro-7H-pyrrolo[2,3-*d*]pyrimidin-4-yl) piperidin-4-yl)-1H-benzo[*d*]imidazole-5-carboxylate (R10015).

There was a 25% yield of R10015 in 4 steps after HPLC purification. ^1^H-NMR (DMSO-*d*_6_, 400 MHz) δ 12.39 (br, 1H), 8.34 (s, 1H), 8.28 (s, 1H), 8.27 (dd, *J* = 1.2, 8.4 Hz, 1H), 7.87 (d, *J* = 8.8 Hz, 1H), 7.59 (d, *J* = 2.4 Hz, 1H), 4.31 to 4.47 (m, 2H), 3.91 (s, 3H), 3.51 to 3.58 (m, 1H), 3.29 (t, *J* = 12.0 Hz, 2H), 2.25 to 2.34 (m, 2H), 2.11 to 2.18 (m, 2H). Analytical HPLC purity: single peak observed at UV = 254 nm; HRMS (ESI-Orbitrap). Calculated for C_20_H_20_ClN_6_O_2_: 411.1336 [M+H^+^]. Found: 411.1328.

### LIMK1 biochemical assay.

Biochemical LIMK1 assays for all inhibitors were carried out by Reaction Biology Corporation, Malvern, PA, and followed the protocols described on its website. The compounds were tested in a 10-dose IC_50_ mode with 3-fold serial dilutions starting at 10 μM. The control compound, staurosporine, was tested in 10-dose IC_50_ mode with 3-fold serial dilutions starting at 10 μM. Reactions were carried out at 10 μM ATP, 1 μM substrate (cofilin), and 50 nM LIMK1 (final concentrations). The specific information for LIMK1 is as follows: GenBank accession number, NP_002305; recombinant catalytic domain, amino acids 285 to 638 (His tagged; purified from insect cells), activated by coexpression of ROCK1. The reagents were as follows: base reaction buffer, 20 mM HEPES (pH 7.5), 10 mM MgCl_2_, 1 mM EGTA, 0.02% Brij 35, 0.02 mg/ml bovine serum albumin (BSA), 0.1 mM Na_3_VO_4_, 2 mM dithiothreitol (DTT), 1% DMSO. No additional cofactors were added to the reaction mixture. The reaction procedures were as follows. (i) The indicated substrate was prepared in freshly made base reaction buffer. (ii) Any required cofactors were delivered to the substrate solution described above. (iii) The indicated kinase was delivered into the substrate solution and gently mixed. (iv) The compounds in DMSO were delivered into the kinase reaction mixture. (v) [^33^P]ATP (final specific activity, 0.01 μCi/μl) was delivered into the reaction mixture to initiate the reaction. (vi) The kinase reaction mixture was incubated for 120 min at room temperature. (vii) The reaction mixtures were spotted onto P81 ion-exchange paper (Whatman no. 3698-915; GE Healthcare Bio-Sciences, Pittsburgh, PA). (viii) The filters were washed extensively in 0.1% phosphoric acid.

### R10015 docking studies.

The inhibitor R10015 was prepared for glide docking using LigPrep (Schrodinger, New York, NY). Chain A of PDB accession no. 3S95 was prepared using the protein preparation wizard in Maestro V 9.8 (Schrodinger, New York, NY) by removing water molecules and bound ligand and adding hydrogen atoms. The docking grid was generated around the original ligand with a box size of 18 by 18 by 18 Å. Docking was conducted without any constraint. The top-scored docking pose was merged with the protein for energy minimization using Prime (Schrodinger, New York, NY).

### Isolation of PBMC and resting CD4 T cells from peripheral blood.

All protocols involving human subjects were reviewed and approved by the George Mason University institutional review board. PBMC were purified from peripheral blood of HIV-negative donors by centrifugation in lymphocyte separation medium (Corning), and resting CD4 T cells were further purified by two rounds of negative selection, as previously described (8, 51). Briefly, for the first-round depletion, we used monoclonal antibodies against human CD14; CD56; and HLA-DR, -DP, and -DQ (BD Biosciences). For the second-round depletion, we used monoclonal antibodies against human CD8, CD11b, and CD19 (BD Biosciences, San Jose, CA). Antibody-bound cells were depleted using Dynabeads Pan Mouse IgG (Invitrogen, Carlsbad, CA). For further negative selection of the memory and naive CD4 T cell subsets, monoclonal antibody against either CD45RA (0.02 μl per million cells) or CD45RO (0.1 μl per million cells) (BD Biosciences, San Jose, CA) was added during the second round of depletion. Purified cells were cultured in RPMI 1640 medium supplemented with 10% heat-inactivated fetal bovine serum (FBS) (Invitrogen, Carlsbad, CA), penicillin (50 U/ml; Invitrogen, Carlsbad, CA), and streptomycin (50 μg/ml; Invitrogen, Carlsbad, CA). The cells were rested overnight before infection or treatment.

### Viruses and viral infection.

Virus stocks of HIV-1(NL4-3) and HIV-1(AD8) were prepared by transfection of HeLa or HEK293T cells with cloned proviral DNA, as described previously (8, 51). HIV-1(VSV G) was prepared as described previously (45). The primary isolates, HIV 92/BR/018 (Brazil) and HIV-1 93UG070 (Uganda), were received from the NIH AIDS Reagent Program. Levels of p24 in the viral supernatant were measured in triplicate by enzyme-linked immunosorbent assay (ELISA) using an in-house ELISA kit. The viral titer (50% tissue culture infective dose [TCID_50_]) was determined on the Rev-dependent GFP and luciferase indicator cell line Rev-CEM-GFP-Luc (23). For HIV infection of Rev-CEM-GFP-Luc cells, 2 × 10^5^ cells were treated with LIMK inhibitors for 1 h and then infected with 10^3^ to 10^4.5^ TCID_50_ units of HIV-1 for 3 h, with the addition of LIMK inhibitors to maintain the drug concentration. The infected cells were washed twice and then resuspended in 1 ml fresh medium without the addition of inhibitors. The cells were incubated for 2 days, and viral infection was measured by flow cytometry (FACSCalibur; BD Biosciences, San Jose, CA) for GFP-positive cells. To exclude drug cytotoxicity, propidium iodide (PI) (2 μg/ml; Sigma-Aldrich, St. Louis, MO) was added to the cell suspension prior to flow cytometry, and only viable (PI-negative) cells were used for measuring GFP expression. For the luciferase assay, the cells were resuspended in 100 μl of luciferase assay buffer (Promega, Madison, WI) and measured using the GloMax-Multi detection system (Promega, Madison, WI).

For viral infection of resting CD4^+^ T cells, unless otherwise specified, 10^6^ cells were treated with LIMK inhibitors for 1 h, and 10^3.5^ to 10^4.5^ TCID_50_ units of HIV-1 were used to infect 10^6^ cells. During infection, LIMK inhibitors were added to maintain the drug concentration. The cells were washed once and then resuspended in fresh medium (10^6^ cells per ml) and incubated for 5 days without stimulation. The cells were activated with anti-CD3/CD28 magnetic beads at 4 beads per cell. Culture supernatant (100 μl) was taken every 2 days or daily after stimulation. The cells were removed by centrifugation, and the supernatant was saved for p24 ELISA.

For viral infection of PBMC, cells were cultured for 24 h (10^6^ cells per ml) and then treated with R10015 for 1 h and infected with HIV for 3 h. Following infection, the cells were washed to remove the virus and the drug and cultured in complete RPMI 1640 medium containing interleukin 2 (IL-2) (100 U/ml) and phytohemagglutinin (PHA) (3 μg/ml; Sigma) for 3 days.

HSV-1 was kindly provided by Timothy M. Block, Drexel Institute for Biotechnology and Virology Research. The virus was propagated on Vero cells. Briefly, cells were infected with HSV-1 (multiplicity of infection [MOI], 0.001) until 100% of the cells displayed cytopathic effect (CPE). The cell supernatant with HSV-1 was harvested by centrifugation at 2,000 rpm for 5 min at 4°C, filtered through a 0.45-μm filter, and then stored at −80°C. For HSV-1 infection, Vero cells were seeded in 10-cm petri dishes and cultured overnight. R10015 or DMSO was added to the cells for 2 h. HSV-1 was serially diluted with 199V medium and added to the cells for infection for 2 h. The cells were washed and cultured in fresh medium (Dulbecco's modified Eagle's medium [DMEM] plus 5% fetal calf serum [FBS]; Invitrogen, Carlsbad, CA) containing 7.5 μg/ml pooled human immunoglobulin. Viral plaques were stained by rinsing twice in phosphate-buffered saline with potassium. The cells were fixed with methanol and stained with KaryoMax Giemsa stain solution (Invitrogen, Carlsbad, CA).

VEEV TC83-Luc was kindly provided by Slobodan Paessler of the University of Texas Medical Branch at Galveston, Galveston, TX. RVFV MP12-luc was kindly provided by Shinji Makino of the University of Texas Medical Branch at Galveston. Venezuelan equine encephalitis virus Trinidad donkey (subtype IA/B), NR-332, was obtained through the NIH Biodefense and Emerging Infections Research Resources Repository, NIAID, NIH. For infections with VEEV-Luc (TC83), VEEV (TC83) (BSL2 strain), VEEV (TrD) (BSL3 strain), or RVFV-Luc (MP12), Vero cells were pretreated with R10015 or DMSO, infected with the viruses at an MOI of 0.1, and posttreated with R10015. The viral supernatants were collected 24 h postinfection and analyzed by plaque assays. Alternatively, for luciferase-expressing viruses, luciferase activity was assessed at 24 h postexpression, and DMSO-treated samples were set to 100%. For EBOV (Zaire) infection, HFF-1 cells were pretreated with R10015 for 2 h and infected with EBOV (Zaire) (MOI, 2.5). Infection was terminated at 48 h postinfection, and the cells were fixed with formalin solution. Infected cells were identified by immunostaining of the EBOV GP protein with a primary mouse antibody and a secondary Alexa488-labeled anti-mouse IgG antibody. The cells were also stained with Hoechst (Invitrogen) for nuclei and with Cell Mask cytoplasm stain (Invitrogen, Carlsbad, CA) for cytoplasm. The number of nuclei per well was used to determine cell viability. Images were taken with a PE Opera confocal platform with 10× objectives and analyzed using Acapella and GeneData software.

### Animal experiments.

Six- to 8-week-old female C3H/HeN mice were obtained from Harlan Laboratories. Groups of 3 mice were treated once a day with compounds as follows: R10904, R10906, R10907, or R7826 delivered via oral gavage at 20 mg/kg; PBS by oral gavage as a control; R10015 (dissolved in DMSO) by i.p. injection at 10 mg/kg; or DMSO by i.p. injection as a control. The mice were weighed daily and monitored for morbidity and mortality, including lethargy and ruffled fur, for 7 days. Experiments were carried out in animal BSL2 facilities in accordance with the National Research Council's Guide for the Care and Use of Laboratory Animals and under GMU IACUC protocol number 0211.

### Western blotting for LIMK, cofilin, and PAK2.

One million cells were lysed in NuPAGE LDS sample buffer (Invitrogen, Carlsbad, CA), separated by SDS-PAGE, and then transferred onto nitrocellulose membranes (Invitrogen, Carlsbad, CA). The membranes were washed in TBST (137 mM NaCl, 2.7 mM KCl, 19 mM Tris base, 0.1% Tween 20) for 3 min and then blocked for 30 min at room temperature with Starting Block blocking buffer (Thermo Fisher Scientific, Grand Island, NY). For probing with different antibodies, blots were incubated with rabbit anti-p-LIMK1/2 antibodies (Cell Signaling, Danvers, MA), with rabbit anti-LIMK1 antibodies (Cell Signaling, Danvers, MA), with rabbit anti-p-cofilin antibodies (Cell Signaling, Danvers, MA), with rabbit anti-cofilin antibodies (Cell Signaling, Danvers, MA), or with rabbit anti-PAK2 antibody (Cell Signaling, Danvers, MA). These antibodies were diluted in 2.5% milk-TBST and incubated with the blots overnight at 4°C. The blots were washed three times for 15 min each time, incubated with anti-rabbit horseradish peroxidase-conjugated secondary antibodies (KPL, Gaithersburg, MD; 1:5,000) for 1 h, and then developed with SuperSignal West Femto Maximum Sensitivity substrate (Thermo Fisher Scientific, Grand Island, NY). For loading control, the same blots were stripped and reprobed with antibodies against GAPDH (glyceraldehyde-3-phosphate dehydrogenase) (Abcam, Cambridge, MA; 1:1,000). Images were captured with a charge-coupled-device (CCD) camera (Protein Simple, San Jose, CA).

### Intracellular p-cofilin staining and flow cytometry.

One million cells were fixed, permeabilized with methanol, washed twice, and then stained with rabbit anti-human p-cofilin antibodies (Cell Signaling, Danvers, MA) for 60 min at room temperature. The cells were washed twice and stained with 10 μg/ml Alexa Fluor 647 (Abcam, Cambridge, MA)-labeled chicken anti-rabbit antibodies (Invitrogen, Carlsbad, CA). The cells were washed twice and then analyzed on a FACSCalibur (BD Biosciences, San Jose, CA).

### Surface staining of CD4 and CXCR4.

Cells were stained with FITC-labeled monoclonal antibody against human CD4 (clone RPA-T4) or CXCR4 (clone 12G5) (BD Biosciences, San Jose, CA). The cells were stained on ice in PBS plus 0.1% BSA for 30 min, washed with cold PBS-0.5% BSA, and then analyzed on a FACSCalibur (BD Biosciences, San Jose, CA).

### Quantitative real-time PCR.

HIV DNA was quantified using the Bio-Rad iQ5 real-time PCR detection system, utilizing the forward primer 5′LTR-U5, the reverse primer 3′ gag, and the probe FAM-U5/gag, as previously described (8). The prequalified, full-length proviral plasmid pNL4-3 was used as the DNA standard. Viral 2-LTR circles were measured as described previously (8). For measuring 2-LTR circles, the DNA was amplified by real-time PCR with primers MH536 and MH535 and probe MH603 (8).

### Chemotaxis assay.

A half million Jurkat T cells were resuspended in 100 μl RPMI 1640 medium and then added to the upper chamber of a 24-well transwell plate (Corning, Corning, NY). The lower chamber was filled with 600 μl of medium premixed with SDF-1 (40 ng/ml). The plate was incubated at 37°C for 2 h, and then the upper chamber was removed and the cells in the lower chamber were counted. Where indicated, different concentrations of R10015 were added to the culture supernatant prior to the assay, along with a DMSO control.

### FITC-phalloidin staining of F-actin and flow cytometry.

F-actin staining using FITC-phalloidin (Sigma-Aldrich, St. Louis, MO) was carried out using 1 × 10^6^ cells. The cells were pelleted, fixed, and permeabilized with CytoPerm/Cytofix buffer (BD Biosciences, San Jose, CA) for 20 min at room temperature and washed with cold Perm/Wash buffer (BD Biosciences, San Jose, CA) twice, followed by staining with 5 μl of 0.3 mM FITC-labeled phalloidin (Sigma-Aldrich, St. Louis, MO) for 30 min on ice in the dark. The cells were washed twice with cold Perm/Wash buffer, resuspended in 1% paraformaldehyde, and analyzed on a FACSCalibur (BD Biosciences, San Jose, CA).

### Viral entry assays.

The BlaM-Vpr-based viral entry assay was performed as previously described (8, 46). We also used a Nef-luciferase-based entry assay as described previously (47). Briefly, cells (1 × 10^6^) were treated with 100 μM R10015 for 1 h, infected with 200 ng of Nef-luciferase-containing viruses at 37°C for 3 h with the same R10015 concentration, and then washed three times with medium. The cells were resuspended in 100 μl of luciferase assay buffer (Promega, Madison, WI), and luciferase activity was measured in live cells using a GloMax-Multi detection system (Promega, Madison, WI).

### Viral release assay.

Chronically HIV-infected ACH2 cells were obtained from the NIH AIDS Reagent Program. The cells were cultured in RPMI 1640 (Invitrogen) plus 10% FBS. The cells were washed 3 times prior to treatment with R10015. DMSO was used as a control. The culture supernatants were harvested on days 2 and 3 post-R10015 treatment and analyzed for HIV-1 p24 by ELISA. For quantification of virion release from DNA-transfected HEK293 cells, the cells were transfected with pHIV(NL4-3) using Lipofectamine 2000 (Thermo Fisher Scientific). After 5 h, the cells were washed and cultured in complete DMEM and then treated with different concentrations of R10015 for 48 h. DMSO was used as a control. The supernatants were harvested and analyzed by ELISA for HIV-1 p24.

### Surface staining of CD25 and CD69.

A half million resting CD4 T cells were cultured for 5 days and stimulated with anti-CD3/CD28 beads (4 beads per cell) for 24 h. The cells were stained with phycoerythrin (PE)-labeled monoclonal antibody against human CD25 (clone M-A251) or CD69 (clone FN50) (BD Biosciences, San Jose, CA). The cells were stained on ice in PBS plus 0.1% BSA for 30 min, washed with cold PBS-0.5% BSA, and then analyzed on a FACSCalibur (BD Biosciences, San Jose, CA).

### Cytotoxicity assay.

The cytotoxicity assay was performed using the MultiTox-Glo Multiplex cytotoxicity assay kit (Promega), as suggested by the kit manufacturer. Cells were cultured in a Cellstar white 96-well plate (Greiner) at a density of 15,000 cells/well (CEM T cells) or 5,000 cells/well (293T cells) and then treated with R10015 for various times at various drug concentrations. DMSO was used as the control. For toxicity control, 100 μl of 1% saponin was added to cells for 3 h to induce cell lysis. Cytotoxicity is presented in relative luminescence units as measured by the GloMax Discover System plate reader (Promega).

### Confocal microscopy.

Cells were imaged using a Carl Zeiss Zen 780 laser scanning microscope with a 40×/numerical aperture (NA) Oil DIC Plan-Apochromatic objective in the green fluorescence channel. The images were then processed and analyzed with Zen 780 software.
